# Incidence and Risk Factors of Wound Infection in Women Who Underwent Cesarean Section in 2014 at King Abdulaziz Medical City, Jeddah

**DOI:** 10.7759/cureus.12164

**Published:** 2020-12-19

**Authors:** Roaa Gadeer, Nada Y Baatiah, Nourah Alageel, Mohammed Khaled

**Affiliations:** 1 Department of Obstetrics and Gynecology, King Faisal Specialist Hospital & Research Centre, Jeddah, SAU; 2 Clinical Nutrition, King Saud Bin Abdulaziz University for Health Sciences, Jeddah, SAU; 3 Medicine, King Saud Bin Abdulaziz University for Health Sciences, Jeddah, SAU; 4 Consultant Obstetrics and Gynecology, The Ministry of National Guard Health Affairs, Jeddah, SAU

**Keywords:** cesarean section, wound infections, emergency and elective cesarean, surgical site infection

## Abstract

Introduction

Cesarean section (C/S) is considered one of the most commonly performed procedures among women. The maternal morbidity due to infection post-C/S reaches eight-fold higher than that of vaginal delivery. Our aim is to identify the incidence and risk factors of surgical site infection (SSI) among patients at King Abdul Aziz Medical City (KAMC), Jeddah, Saudi Arabia, in order to develop a strong strategy to reduce its occurrence.

Methods

This retrospective cohort study was conducted at KAMC, Jeddah. The study included a total of 387 women who underwent cesarean sections from January 2014 to December 2014. The data were collected consecutively by reviewing medical records of pregnant patients who underwent elective or emergency C/S. The risk factors studied included age, presence of underlying diseases, BMI, hemoglobin level, prophylactic antibiotics, pre-labor rupture of membrane, duration of induction of labor, type of C/S, type of uterine incision, duration of operation, type of anesthesia, estimated blood loss, type of organism, and the duration of hospital stay postoperatively.

Results

The incidence rate of wound infections (WI) was 3.4% (13/387). The mean age score was 31.1±5.6 years, and the mean score of BMI was 32.7±6.2, where the majority were obese (255, 65.9%). More than half of the participants (205, 53.0%) had elective C/S, with mean hospitalization duration 2.5±1.3 days, and operation duration mean score 59.5±22.0 minutes. The majority (378, 97.7%) received antibiotics before the operation, where cefazolin was the main antibiotic (376, 97.2%). Only 38 (10%) cases had intra-operative complications, where the main complication was postpartum hemorrhage (18, 44.0%). The majority of WI were superficial (11 cases), the main organism was *E. coli* in four (36.4%) cases, followed by *Staphylococcus aureus* in three (27.3%) cases. There was a significant association between WI post-C/S and BMI, type of uterine incision, and induction of labor (P=006, P=0.003, respectively).

Conclusions

This study showed that WI post-C/S is associated with high BMI, prolonged induction of labor, and Pfannenstiel incision. Reducing the rate of SSI will help to reduce its morbidity by identifying the risk factors pre-pregnancy and encouraging the implementation of preconception counseling clinics and antenatal classes to educate and increase awareness among patients.

## Introduction

Cesarean section (C/S) is considered one of the most commonly performed procedure among women [1]. The C/S rate has increased over the years, and the international rate reached approximately 15% [2]. A study showed that the maternal morbidity due to infection post-C/S reached eight-fold higher than vaginal delivery [3]. Also, studies indicate that surgical site infection (SSI) post-C/S has the highest number among infections [4,5]. According to the Centers for Disease Control and Prevention (CDC), SSI refers to any infections occurring within 30 days after surgical operation [6]. SSIs are common surgical complications among patients who delivered with C/S. A study at the university hospital in Jeddah reported an SSI rate of 19% after cesarean delivery [7]. As a result of having SSI, the duration of the hospital stay and the rate of hospital re-admission will increase, also the rate of mortality and morbidity will become higher [8]. According to the CDC, SSI has been classified into subgroups; the first subgroup is superficial incisional SSI, which involves only skin or subcutaneous tissue. The second subgroup is deep incisional SSI, which involves deep soft tissues of the incision and organ. The last subgroup is space SSI that involves any part of the anatomy [9]. 

There are many risk factors for SSI post-C/S. They can be divided into intrinsic (patient-related) and extrinsic (management and care-related) risk factors. An example of the intrinsic factor is the age of the patient; pregnant patients who are 35 and above are at a higher chance to develop SSI post-C/S [10]. Another risk factor is obesity; pregnant patients with a high body mass index (BMI) (25 and above) have a higher risk for SSI after C/S [11]. Also, pregnant patients with other co-morbidities such as diabetes mellitus, anemia, and hypertension have more risk to have SSI post-C/S [12]. Regarding the extrinsic factors, one of the risk factors is the inappropriate use of antibiotics. Patients who received an inappropriate type of antibiotics or received the dose at an inappropriate time had an increased risk of SSI post-C/S [13]. The second risk factor is the number of vaginal or anal examinations. If the patient had multiple vaginal or anal examinations, then the patient is at higher risk to have SSI after C/S. Another risk factor is blood loss; pregnant patients who had excessive blood loss during the surgery had a higher risk to have SSI post-C/S [11]. Also, operative time is one of the risk factors of having SSI post-C/S. If the duration of the operation took more than one hour, the patients will be at more risk to have SSI [11]. Patients who had prolonged labor or had pre-labor rupture of the membrane more than 24 hours are at higher risk to develop SSI [8]. Moreover, emergency C/S is another risk factor of SSI. The patients who underwent emergency C/S are at high risk to have SSI [8]. Also, the character and bacteriological study of wound discharge are considered as a risk factor of SSI. By wound swab, studies found that most of the patients their infection occurred by *Staphylococcus* [8]. Another important risk factor is the type of incision. Many studies found that Pfannenstiel (transverse) incision has more risk to develop SSI post-C/S [10]. Also, if the anesthesia was spinal, the risk to develop SSI after C/S is higher [14]. Finally, the skill level of the surgeon is considered a risk factor of having SSI post-C/S. If the surgeon is a junior, the risk to have SSI will be higher [15].

There are a lot of studies that report the incidence and risk factors of wound infection (WI) in women who underwent C/S internationally, but we could not find any study that talks about risk factors of SSI post-C/S in Saudi Arabia. Also, there is only one old study, which mentioned the incidence of SSI post-C/S at King Abdul-Aziz hospital in Jeddah in 1992 [7]. 

The study will increase the public data available regarding the risk factors of WI in women who undergo C/S in Saudi Arabia. We aim to identify the incidence and the significant risk factors of SSI during 2014 among patients at King Abdulaziz Medical City (KAMC), Jeddah, in order to help in developing strong strategies to reduce its occurrence.

## Materials and methods

In this retrospective cohort study, the data was collected consecutively by reviewing medical records of pregnant patients who underwent elective or emergency C/S at KAMC, Jeddah in the study period from January 2014 till December 2014. Three hundred eighty-seven cases were reviewed to compare the incidence of WI among women who underwent emergency C/S with those who underwent elective C/S in all ages. The study was approved by King Abdullah International Medical Research Center (IRBC/1417/17).

The data were collected by research team members through a standard data collection tool design for this study. Patients' demographics, presence of underlying diseases, preoperative, intra-operative, and postoperative data were compared among these patients. The preoperative data include hemoglobin level and prophylactic antibiotics. Intra-operative data include the type of uterine incision, duration of the operation, type of anesthesia, and estimated blood loss. Postoperative data include the duration of hospital stay postoperatively, SSI, and its classification. Completed data collection sheet transferred to excel sheet in a secured computer. The data encrypted and stored in a locked file accessed only by the primary investigator. Data have been reviewed and cleaned before analysis.

As a policy of the hospital, all patients who underwent C/S at KAMC should receive prophylaxis antibiotics of 1 gram, intravenous cefazolin, pre-operational, unless the patient is allergic to it (clindamycin or vancomycin will be given). The scrubbing used to clean the patient is standardized, using chlorhexidine and the scrubbing used by the doctors is chlorhexidine digluconate 4%.

SSI was classified according to the CDC classification: superficial infection that involves only skin and subcutaneous tissue, deep infection that involves deep tissue (fascial and muscle layers), and organ/space infection that involves any part of the body deeper than the fascial/muscle layers. Moreover, the World Health Organization (WHO) classification was used for BMI categories: underweight < 18.5, normal range (18.5-24.9), overweight (25-29.9), obese >= 30.

Statistical analysis was performed using Statistical Package for Social Sciences (SPSS) version 22 (IBM Corp., Armonk, NY, USA). Qualitative variables were presented using frequencies and percentages while quantitative variables were presented by the mean and standard deviation. To assess the significance of the relationship between the risk factors variables, the type of C/S (elective or emergency), and the development of the outcome, which is SSI, we applied a Chi-square test, two-sided, with the level of a significant set at 0.05. Chi-square test for categorical data and multivariable regression models performed in order to identify factors associated with SSI post-C/S. 

## Results

This study enrolled 387 C/S performed in the study period from January 2014 to December 2014. The incidence rate of WI was 3.4% (13/387). The mean age score was 31.1±5.6 years ranging from 19 to 51 years. The mean score of BMI was 32.7±6.2, where the majority were obese (255, 65.9%) or overweight (97, 25.1%). A quarter of the cases were diabetic patients (25.3%), out of which 90 (23.2%) cases had gestational diabetes. The median scores of gravidity, parity, and previous C/S were 3±3, 2±2, and 2±2 respectively. 

The results showed that the mean score of gestational age was 38.5±2.2 weeks, 19 (4.9%) cases had multiple pregnancies, and 80 (20.7%) had pre rupture of membranes (PROM). Also, 68 (17.6%) patients had meconium-stained liquor, and only three (0.8%) had chorioamnionitis. More than half of the cases (205, 53%) had elective C/S, with mean hospitalization score 2.5±1.3 days, and operation duration mean score 59.5±22.0 minutes. The transverse incision was the type of uterine incision in 383 (99.0%) of the cases. The mean level of hemoglobin before the operation was 11.5±1.3 g/dl and the average estimated blood loss was 815±382 ml. The majority of patients (378, 97.7%) received antibiotics before the operation, where cefazolin was the main used (376, 97.2%), and the other two cases received a combination of antibiotics. Only 38 (10%) cases had intra-operative complications, where the main complication was postpartum hemorrhage (18, 44.0%) followed by extension (11, 26.8%), then bowel injury (five, 12.2%). Group B strep tests (GBS) status were positive in 49 (12.7%) and negative in 112 (29%). Two-thirds of the cases (255, 65.9%) received spinal anesthesia, followed by epidural (72, 18.6%), then general (58, 15.0%). Moreover, 70 (18.1%) reported induction of labor, and only two (0.5%) cases received immunosuppressive medication. The additional pregnancy information is presented in Table 1. 

**Table 1:** Current pregnancy information C/S: Cesarean section; GBS: group B strep; PPH: postpartum hemorrhage; IVIG: intravenous immunoglobulin

Variable	N	%
Experience multiple pregnancies:		
Yes	19	4.9
No	368	95.1
Having rupture of membrane:		
Yes	80	20.7
No	307	79.3
Having meconium-stained liquor:		
Yes	68	17.6
No	319	82.4
Chorioamnionitis:		
Yes	3	.8
No	384	99.2
Type of caesarean section:		
Emergency caesarean section	182	47.0
Elective caesarean section	205	53.0
Type of uterine incision:		
Classical	4	1.0
Transverse	383	99.0
Antibiotic received pre-operation:		
Yes	378	97.7
No	9	2.3
Type of antibiotic received		
Not applicable	9	2.3
Cefazolin	376	97.2
Ampicillin, Gentamysin, Metramideole	1	0.3
Gentamysin,Clindamycin	1	0.3
Having intr-aoperative complications:		
Yes	38	10.0
No	349	90.0
What is the complication?		
Ureter ligation	1	2.4
Bowel injury	5	12.2
Extension	11	26.8
PPH	18	44.0
Scar dehiscence	1	2.4
Partial Placenta accreta	1	2.4
Placenta accreta	1	2.4
Bilateral internal illiac atery ligation	1	2.4
Ureteric Injury	2	4.8
GBS status:		
Positive	49	12.7
Negative	112	29.0
Unknown	226	58.3
Type of anesthesia:		
Epidural	72	18.6
Spinal	255	65.9
General	58	15.0
Unknown	2	0.5
Level of training (Surgeon):		
Seniors	329	85.0
Residents	54	14.0
Induction of labor:		
Yes	70	18.1
No	317	81.9
taking immunosuppressive medications:		
Yes	2	.5
No	385	99.5
Name of immunosuppressive used		
Hydrocortisone & Prednisolon	1	50
Prednisolon , Hydrocortisone , IVIG	1	50
Variables	Mean± SD	Range (min-max)
Gestational age:	38.5±2.2 weeks	(29-42)
C/S Duration of hospital stay after C/S:	2.5±1.3 days	(1-21)
Preoperative hemoglobin level:	11.5±1.3 g/dl	(7.2-15.3)
Duration of the surgery:	59.5±22.0 minutes	(17-240)
Blood loss:	815±382 ml	(300-4500)

Only 13 (3.4%) cases suffered from WI, where the majority of WI were superficial (11 cases). The main organism was *Escherichia coli* in four (36.4%) cases, followed by *Staphylococcus aureus* in three (27.3%) cases. Only two (0.5%) cases required readmission due to WI, with mean length of stay 7.5±2.2 days, as can be seen in Table 2.

**Table 2:** Post Cesarean section (C/S) and wound infection data

Variable	N	%
Developing wound infection:		
Yes	13	3.4
No	374	96.6
Classification of wound infection:		
Superficial	11	84.6
Deep	1	7.7
Unknown	1	7.7
Type of the organism:		
Staph. Aureus	3	27.3
Unknown	3	27.3
E.coli	4	36.4
Proteus mirabilis	1	9.1
Enterococcus faecalis	1	9.1
Readmission due to wound infection:		
Yes	2	0.5
No	385	99.5
Complication of wound infection:		
Yes	2	15.4
No	11	84.7

The results in this study revealed a significant association between WI post-C/S and some risk factors include BMI, type of uterine incision, and induction of labor, as illustrated in Table 3. 

**Table 3:** Relation between wound infectious and several risk factors: GDM: gestational diabetes mellitus; DM: diabetes mellitus

Variables	wound infections (Yes) N (%) or Mean± SD	wound infections (No) N (%) or Mean± SD	P-value
BMI:			0.05
Underweight	0	4 (1.1)	
Normal	0	31(8.2)	
Overweight	1 (7.7)	96 (25.7)	
Obesity	12 (92.3)	243 (65.0)	
Meconium:			0.18
Yes	4 (30.8)	64 (17.1)	
No	9 (69.2)	310 (82.9)	
Caesarean section type:			0.41
Emergency	7 (53.8)	175 (46.8)	
Elective	6 (46.2)	199 (53.2)	
Uterine incision:			0.006*
Yes	2 (15.4)	2 (0.5)	
No	11 (84.6)	372 (99.5)	
Received preoperative antibiotic:			0.24
Yes	12 (92.3)	366 (98.1)	
No	1 (7.7)	7 (1.9)	
Developed intraoperative complications:			0.53
Yes	2 (15.4)	36 (9.7)	
No	11(84.6)	337 (90.3)	
Level of training of the Surgeon:			0.43
Senior	12 (92.3)	317 (85.7)	
Resident	1 (7.7)	53 (14.3)	
Underlying diseases:			0.28
GDM	2 (33.3)	88 (62.0)	
DM	1 (16.7)	7 (4.9)	
Other	3 (50.0)	47 (33.1)	
Induction of labor:			0.003*
Yes	7 (53.8)	64 (17.1)	
No	6 (46.2)	310 (82.9)	
Hospitalization duration:	Mean± SD 3.1±1.1	Mean± SD 2.5±1.1	0.10
Operation duration:	Mean± SD 61.8±21.8	Mean± SD 59.4±22.5	0.70

## Discussion

The findings of this study are in agreement with the results in some previous studies. Regarding the incidence of WI, Schneid-Kofman retrospectively analyzed 19,416 patients who had cesarean delivery and found that 726 (3.7%) patients developed WI. Comparing the previous study to our results, the rate of WI post-C/S is 3.4 % [16]. Regarding the risk factors, we found some risk factors that associate with SSI post-C/S and have similar findings with other studies. One of the most significant risk factors is obesity. In a study conducted in China, a total of 13,798 women underwent C/S and the results showed that 96 (0.7%) developed SSI and only 73 cases from the 96 had complete data on risk factors, out of which more than half of them were obese (67.1%) [11]. This finding is similar to our study, which found that the risk of SSI was highest among patients who have higher BMI in the obese range. Moreover, from the data obtained in this study, the mean BMI was 32 with a range from 18-62, which is well above the standard known. This is in itself merits further research. There are various mechanisms that make obesity a very serious risk factor for SSI. In the operating room, obese patients present technical challenges that often result in increased procedure time and resulted in many complications. As a result, the redundant skin over the wound site, sometimes poor hygiene, and poor penetration of antibiotics in the adipose tissue attributed to this associated risk.

Another risk factor is the duration of the induction of labor, which was significantly associated with SSI. In the current study, 53.8% of infected mothers also had a long induction of labor. Likewise, in a cross-sectional observational study aimed to assess the risk factors of incisional SSI, 35% of the women who had SSI post-C/S had prolonged labor [8]. Regarding the bacteriological study, according to the National Nosocomial Infections Surveillance System, *Staphylococcus aureus* accounts for approximately 20% of nosocomial Infections. Furthermore, it is the most common organism isolated from SSI [17]. However, in our study, *Escherichia coli* was isolated in four cases out of 13. Although higher than other studies, the small number may falsely give this impression. 

Another important risk factor that can lead to SSI post-C/S is the level of training. The experience of the consultants and registrars usually protects against WI, but in our study the results were different. We observed that most of the cases of WI were done by seniors, either consultants or registrars. This can be explained by the high-risk cases with high BMI, severe pre-eclampsia, and the second stage of labor usually have been done by the seniors. As a result, that may contribute to a high rate of wound infection in this group.

Regarding the type of incision and its impact in causing SSI post-C/S, Molla retrospectively analyzed 334 women who underwent C/S and had SSI. The study found that 291 (87.1%) of women who had SSI post-C/S had Pfannenstiel incision [10]. Our finding was consistent with this study that 84.6% of women who had C/S and Pfannenstiel incision also found to have SSI. Moreover, another risk factor of SSI post-C/S is the type of anesthesia. Jasim retrospectively analyzed 400 women who underwent C/S and found that 75 (18.8%) had SSI; 58 out of 75 (77.3%) had spinal anesthesia [14]. Our study shows similar findings, that spinal anesthesia is considered a risk of devolving SSI post-C/S. For the other risk factors, although by regression analysis we could not find a significant association between SSI and the other proposed risk factors, which are inappropriate use of prophylactic antibiotics, level of training, numbers of vaginal or oral examinations, excessive blood loss (need further analysis), operation time, duration of hospital stay postoperatively, pre rupture of membrane, emergency C/S, age, and having other comorbidities, but this could be because of the small sample size. Accordingly, multi-center studies are needed. 

## Conclusions

This study showed that WI post-C/S is associated with high BMI, prolonged induction of labor, and Pfannenstiel incision. 

Although the policy of the hospital states clearly that all patients going to the operative room should receive antibiotics (cefazolin 1 g, IV, stat, on-call to operation room unless allergic they will receive clindamycin or vancomycin), it seems that some cases did not receive it and no reason was given. We recommend the infectious and prevention control administrative to emphasize strictly applying this policy.

Reducing the rate of SSI will help to reduce morbidity by identifying the risk factors pre-pregnancy and encouraging implementing preconception counseling clinic and antenatal classes to educate and increase awareness among patients.
